# Avian viral surveillance in Victoria, Australia, and detection of two novel avian herpesviruses

**DOI:** 10.1371/journal.pone.0194457

**Published:** 2018-03-23

**Authors:** Jemima Amery-Gale, Carol A. Hartley, Paola K. Vaz, Marc S. Marenda, Jane Owens, Paul A. Eden, Joanne M. Devlin

**Affiliations:** 1 Asia-Pacific Centre for Animal Health, Melbourne Veterinary School, Faculty of Veterinary and Agricultural Sciences, The University of Melbourne, Parkville, Victoria, Australia; 2 Australian Wildlife Health Centre, Healesville Sanctuary, Zoos Victoria, Badger Creek, Victoria, Australia; 3 Asia-Pacific Centre for Animal Health, Melbourne Veterinary School, Faculty of Veterinary and Agricultural Sciences, The University of Melbourne, Werribee, Victoria, Australia; Linnaeus University, SWEDEN

## Abstract

Viruses in avian hosts can pose threats to avian health and some have zoonotic potential. Hospitals that provide veterinary care for avian patients may serve as a site of exposure of other birds and human staff in the facility to these viruses. They can also provide a useful location to collect samples from avian patients in order to examine the viruses present in wild birds. This study aimed to investigate viruses of biosecurity and/or zoonotic significance in Australian birds by screening samples collected from 409 birds presented to the Australian Wildlife Health Centre at Zoos Victoria’s Healesville Sanctuary for veterinary care between December 2014 and December 2015. Samples were tested for avian influenza viruses, herpesviruses, paramyxoviruses and coronaviruses, using genus- or family-wide polymerase chain reaction methods coupled with sequencing and phylogenetic analyses for detection and identification of both known and novel viruses. A very low prevalence of viruses was detected. *Columbid alphaherpesvirus 1* was detected from a powerful owl (*Ninox strenua*) with inclusion body hepatitis, and an avian paramyxovirus most similar to *Avian avulavirus 5* was detected from a musk lorikeet (*Glossopsitta concinna*). Two distinct novel avian alphaherpesviruses were detected in samples from a sulphur-crested cockatoo (*Cacatua galerita*) and a tawny frogmouth (*Podargus strigoides*). Avian influenza viruses and avian coronaviruses were not detected. The clinical significance of the newly detected viruses remains undetermined. Further studies are needed to assess the host specificity, epidemiology, pathogenicity and host-pathogen relationships of these novel viruses. Further genome characterization is also indicated, and would be required before these viruses can be formally classified taxonomically. The detection of these viruses contributes to our knowledge on avian virodiversity. The low level of avian virus detection, and the absence of any viruses with zoonotic potential, suggests low risk to biosecurity and human health.

## Introduction

Avian species serve as hosts for a diversity of viruses that pose both biosecurity and zoonotic risks. Numerous outbreaks of disease in collections of caged birds and commercial domestic poultry have been attributed to viruses introduced by direct or indirect contact with wild birds [1–7]. Some avian viruses can also cause disease in humans [8–14]. As such, understanding the viruses present in wild birds is important for managing potential risks to human and animal health, and for understanding the biology of these viruses in their natural hosts. Studying viruses in wild birds can be challenging as gaining access to these birds for the purpose of sample collection can be difficult, and their capture may be a cause of stress or injury. Collecting and testing samples from avian patients presenting to veterinary hospitals can be a convenient and useful approach to detecting and studying avian viruses. Importantly this approach does not involve additional capturing or handling of birds, and allows the risks to staff and other avian patients/collections within the facility to be more accurately assessed, since the species from which the samples are collected reflects the species being handled at the participating facility. This approach has been successful in previous studies utilizing veterinary hospitals and wildlife rescue centres in several countries including Australia, the United Kingdom, France and Belgium [15–18].

In Australia, the Australian Wildlife Health Centre (AWHC) at Zoos Victoria’s Healesville Sanctuary functions as a large primary care and referral centre for sick, injured and orphaned native wildlife, including providing veterinary care for approximately 700–900 wild birds each year, as well as Healesville Sanctuary’s collection of captive animals. Patients may be presented to the AWHC by members of the public, wildlife carers, keepers, government wildlife officers or by referral from other veterinary clinics for veterinary diagnosis and treatment. During this period in captivity there is an elevated risk of stress-induced shedding of pathogens, presenting a risk for both the veterinary staff and other animals in care, including the Sanctuary’s collection animals and captive breeding programs. Close contact between infected and susceptible animals can facilitate the transmission of pathogens between species, particularly when the hosts are taxonomically related. The large number of avian patients passing through the AWHC provides an opportunity to study the viruses present in Australian birds and better understand the potential risks these viruses present.

Of particular importance and conservation value at Healesville Sanctuary are the captive breeding and insurance programs for threatened species. This includes a captive breeding program for the critically endangered orange-bellied parrot (*Neophema chrysogaster)*, which is facing imminent extinction in the wild [19]. The orange-bellied parrot is arguably the most threatened parrot species in the world, with the wild-bred population declining to only three females and 13 male birds in the 2016/2017 breeding season [19]. Infectious diseases, including viral diseases, are a recognized threat to this and other threatened bird species both within Australia and around the world. Beak and feather disease virus (BFDV), a circovirus, is of particular concern [20]. Our past work has shown that BFDV is prevalent in both psittacine and non-psittacine birds presenting to the AWHC, and has revealed that non-psittacine birds may represent a previously unrecognized risk for spreading BFDV infection [21]. Other viruses are also a potential threat to Psittaciformes in Australia. *Psittacid alphaherpesvirus 1* (PsHV1) is the aetiologic agent of both Pacheco’s disease and mucosal papillomas in Psittaciformes [22]. PsHV1 has not been reported in wild Psittaciformes in Australia [23], but has been detected in Australia in captive green-winged macaws (*Ara chloropterus*) imported from the United Kingdom in the 1990s [23, 24]. It is not known how far the virus has been transmitted from these birds but it is likely to be present in at least some avicultural collections in Australia [23]. The inadvertent introduction of such a virus into the remaining population of a threatened avian species, such as through the *Neophema chrysogaster* recovery program, would be potentially devastating. Avian paramyxoviruses (APMVs) also pose a threat to Australian Psittaciformes. *Avian avulavirus 3* (previously avian paryamyxovirus serotype 3, APMV-3) has been documented causing mortality rates of up to 70% in captive collections of *Neophema* species and other Australian psittacines overseas [25–28]. APMV-3 strains have been isolated from a diversity of avian species in different parts of the world, but has not yet been reported in Australia [25–31]. However we do know that the Australian parrot genus *Neophema* is highly susceptible to APMV-3, with infection frequently resulting in fatal neurological disease [26, 28]. Another avian paramyxovirus, *Avian avulavirus 5* (avian paramyxovirus 5, APMV-5) has caused outbreaks of severe disease with up to 100% mortality in captive budgerigars (*Melopsittacus undulatus*), including an outbreak in a captive budgerigar flock in Queensland in 1972 [25, 32–34].

Few avian viruses represent a risk to veterinary staff and others handling birds, but avian influenza viruses (AIVs) can threaten human health [8, 9, 11–13]. Wild birds, particularly waterfowl, waders and sea birds, are the natural reservoirs of low pathogenicity avian influenza (LPAI) viruses and play important roles in the circulation of AIVs [35–38], with potential for transmission of AIVs both to and from poultry and the potential for mutation to generate highly pathogenic avian influenza (HPAI) viruses [6, 7, 39]. Some AIVs can be transmitted from poultry to humans, and some have caused severe disease or even death in humans [8, 9, 11–13, 40, 41]. Transmission of AIVs from wild birds to commercial poultry has occurred recently in Australia in Victoria, New South Wales and Queensland [10, 42–45]. The Australian National Avian Influenza Wild Bird Surveillance Program has been in place since 2006 but primarily targets Anseriformes (ducks, swans, geese) and Charadriiformes (gulls, terns and shorebirds) [46, 47]. The prevalence of AIV infection in birds presenting to veterinary care facilities, such as the AWHC, has not been as comprehensively studied [15]. In Australia, avian coronaviruses (AvCoVs) are also of particular interest in wild birds because of the presence of variant forms of the poultry pathogen infectious bronchitis virus (IBV) in Australian commercial poultry [48–50]. It is suspected that the variant IBV strains contain genetic material derived from AvCoVs hosted by wild birds, but this requires further investigation [48].

This study aimed to collect samples from birds presenting to the AWHC and use them for the molecular detection and characterization of viruses with potential significance to biosecurity, human health or animal health. Four virus families were targeted in this study: avian influenza viruses, herpesviruses, paramyxoviruses and coronaviruses. Samples were screened using a combination of genus- or family-wide polymerase chain reaction methods coupled with sequencing and phylogenetic analyses for detection and identification of both known and novel viruses. Two novel viruses were detected in this study, hence contributing to our knowledge of avian virodiversity, while the overall low level of virus detection adds to our understanding about the presence or absence of viruses of birds in Australia and their potential risk to avian and human health.

## Materials and methods

### Sample collection

Samples were collected from 409 birds presented to the AWHC at Healesville Sanctuary (37°68'35"S, 145°53'46"E) for veterinary diagnosis and treatment between December 2014 and December 2015 (see S1 Table). This study was approved by the Animal Ethics Committee of the Faculty of Veterinary and Agricultural Sciences of the University of Melbourne (Ethics ID #1413397). Of these 409 birds, 299 were wild avian patients presented to the AWHC for veterinary care, while 110 were captive (part of the Healesville Sanctuary collection). Information regarding the location, date, species and any other available and relevant details about the bird’s history, including reason for admittance, were collected at the time of admission by AWHC staff. A clinical examination was performed by a veterinarian after admission, with any observed clinical signs and diagnostic findings being recorded, including body condition as judged by palpation of pectoral muscle mass. Samples were collected from live avian patients or at necropsy (from birds that had been euthanased or died for reasons unrelated to this study). Most samples collected from live birds were opportunistically taken whilst birds were anesthetised using inhalational isoflurane (Delvet Isoflurane) and oxygen delivered via mask to facilitate clinical examination and diagnostic investigations, and all efforts were made to minimise animal stress and discomfort. Sterile rayon-tipped dry swabs (Copan Italia) were first moistened by dipping in sterile phosphate buffered saline, then used to separately swab the choana and cloaca of 199 live birds (107 wild and 92 captive avian patients). The tips of these swabs were cut into 500 μL of RNAlater^**®**^ (Ambion) and stored at -20°C. For the 210 birds sampled at post-mortem (comprising 192 deceased wild birds and 18 birds originating from the Healesville Sanctuary collection), sterile dry swabs were used to separately swab the trachea and intestine/caecum, cut into 500 μL of RNAlater^**®**^ and stored at -20°C. A piece of liver was also aseptically collected and stored at either -20°C or -80°C. The collection of these liver samples, and their subsequent testing for BFDV, has been previously reported in a separate study [21]. Instruments used for post-mortem sample collection were cleaned and autoclaved between each necropsy. Samples of liver, spleen, kidney, lung, heart, gonad, pancreas, proventriculus, gizzard, small intestine, large intestine (including caecae if present, depending on bird species), pectoral muscle, skin, brain and any lesions were also collected and fixed by submersion in 10% phosphate-buffered formalin at necropsy. For a few select birds of interest, formalin-fixed, paraffin-embedded tissue sections were stained with haematoxylin and eosin for histological examination.

### Nucleic acid extraction

Swabs in RNAlater^®^ were vortexed for 10 seconds and then centrifuged briefly before 100 μL aliquots of each choanal/tracheal and cloacal/intestinal swab in RNAlater^®^ from the same bird were pooled to produce 200 μL samples for genomic nucleic acid extraction. Nucleic acid extraction was performed manually using a QIAamp Viral RNA Mini Kit (Qiagen), using the spin protocol according to the manufacturer’s instructions, eluting into two lots of 40 μL of Buffer AVE, and robotically using VX Universal Liquid Sample DNA Extraction Kits (Qiagen) and a Corbett X-tractor Gene Robot (Corbett Robotics), according to the manufacturer’s instructions, with products being eluted in 70 μL of elution buffer. Samples of liver tissue collected at post-mortem were pulverised with a swab and the swab placed into 500 μL RNAlater^®^. Liver swabs in RNAlater^®^ were vortexed for 10 seconds then centrifuged briefly before taking 200 μL aliquots from which nucleic acid was extracted as above. Whenever pooled clinical samples tested positive by PCR, nucleic acid was extracted from 140 μL aliquots of the corresponding original choanal/tracheal and cloacal/intestinal swab samples separately using the QIAamp Viral RNA Mini Kit spin protocol, and eluted in 60 μL of Buffer AVE. Laboratory stocks of Influenza A/Memphis/1/1971 (H3N2), *Chlamydia felis*, *Macropodid alphaherpesvirus 1* 3076/08 and Newcastle disease virus vaccine strain (NDV.V4) were pooled to give 200 μL positive extraction control samples. Negative extraction control samples utilised 200 μL aliquots of RNAlater^®^.

### PCR detection of avian herpesviruses

Samples were tested for the presence of herpesvirus DNA using a universal herpesvirus PCR assay utilising degenerate, deoxyinosine-substituted oligonucleotide primers specifically targeting a highly conserved region of the herpesvirus DNA polymerase gene, as previously described [51]. *Macropodid alphaherpesvirus 1* or *Equid alphaherpesvirus 4* DNA were used as template for positive control reactions, and sterile H_2_O for negative control reactions. When herpesvirus DNA was detected, additional viral DNA sequence information was obtained by first repeating the first round of the nested universal herpesvirus PCR assay, using DNA extracted from separate choanal/tracheal and cloacal/intestinal swab samples as template. Further PCRs using forward primer TGV paired with reverse primer KG1, and forward primer DFA paired with reverse primer IYG, were then performed using 5 μL aliquots of the first round reaction as template.

### PCR detection of RNA viruses

#### cDNA synthesis

SuperScript™ III Reverse Transcriptase (Invitrogen) was used to synthesise cDNA from aliquots of extracted nucleic acid in preparation for screening of samples for the presence of paramyxoviruses and coronaviruses by PCR. The reverse transcriptase (RT) reaction was performed according to the manufacturer’s instructions using 100 ng random primer oligonucleotides (Invitrogen), and 10 μL of nucleic acid extract as template. All assays included a no template control with sterile water, and the appropriate positive control as described for each assay.

#### Avian paramyxoviruses

Samples were tested for the presence of paramyxovirus RNA using a family-wide RT-PCR assay utilising consensus degenerate and inosine-containing oligonucleotide primers annealing to conserved motifs in domain III of the RNA-dependent RNA polymerase gene, as described previously [52]. cDNA synthesised from RNA extracted from Newcastle disease virus vaccine strain (NDV.V4) was used as the template for positive control reactions. Products amplified by PCR were separated on agarose gels using SYBR Safe (Invitrogen) and visualised by UV transillumination using Bio-Rad Image Lab Software. When *Paramyxoviridae* RNA was detected by RT-PCR, forward and reverse primers PAR-F1 and PAR-R [53] were used in combination with the degenerate oligonucleotide primer pair PMX1 and PMX2 [52] to obtain additional sequence information. Both sets of degenerate primers target the same conserved region of the L-protein-coding sequences of the RNA-dependent RNA polymerase gene–the most conserved viral gene in the family *Paramyxoviridae* [53]. PAR-F1 was coupled with PMX2 to produce amplicons of approximately 120 bp in size, while PMX1 coupled with PAR-R produced amplicons of approximately 650 bp in size. The PCR mixtures contained 0.5 μM of each forward and reverse primer (GeneWorks), 2.5 μL of cDNA template, 200 μM (each) deoxynucleoside triphosphate, Green GoTaq^**®**^ Flexi buffer, 3 mM MgCl_2_ and 1 U GoTaq^**®**^ Flexi DNA Polymerase in a 25 μL reaction volume. PCR mixtures were incubated at 94°C for 2 minutes prior to 40 cycles of 94°C for 15 seconds, 41°C for 30 seconds and 72°C for 30 seconds, followed by a final extension at 72°C for 7 minutes.

#### Avian coronaviruses

A real-time RT-PCR assay using primers targeting conserved sequences within the 5’-untranslated region (UTR) gene and a TaqMan^**®**^ dual-labelled probe [54] was used to screen samples for the presence of avian gammacoronaviruses of the IBV lineage. The 25 μL reactions used TaqMan^**®**^ GTXpress™ Master Mix (Life Technologies) according to the manufacturer’s instructions, with forward and reverse primers to a final concentration of 0.5 μM each, 5-carboxyfluorescein (FAM)-labelled probe with 3’-BHQ1-quencher (GeneWorks) to a final concentration of 0.1 μM, and 5 μL of cDNA template. cDNA synthesised from RNA extracted from laboratory stocks of infectious bronchitis virus (strain VIC-S) was used as the template for positive controls reactions. Reactions were conducted in a Stratagene Mx3000P™ quantitative PCR thermocycler, with initial enzyme activation at 95°C for 20 seconds, then 40 cycles of a denature step at 94°C for 1 second followed by 60°C for 60 seconds for annealing and extension. Results were analysed using Stratagene MxPro software, with C_T_ values above 40 considered negative.

#### Influenza A viruses

Samples were tested for the presence of influenza A virus RNA using a TaqMan Real-Time matrix gene-specific RT-PCT assay [55] within a commercial kit (AgPath-ID™ One-Step RT-PCR Kit, Ambion). RNA extracted from Influenza A/Memphis/1/1971 was used as template for positive control reactions, while sterile water was used for the negative control reactions. Reactions were performed in a Stratagene Mx3000P™ thermocycler. Cycle threshold values above 40 were considered negative.

Throughout this study 95% confidence intervals (95% C. I.) for sample proportions and prevalence estimates were calculated using the Jeffreys method [56], while statistical analysis was carried out using the Fisher exact chi-squared test [57].

### DNA sequencing and phylogenetic analyses

PCR products were purified from PCR reaction mixtures and sequenced as described previously [21]. Geneious^**®**^ 9.1.8 bioinformatics software (Biomatters Ltd., Auckland, New Zealand) [58] was used to trim and align all obtained sequences. Nucleotide sequences were compared with publicly available sequences in the GenBank^**®**^ database (National Center for Biotechnology Information, http://www.ncbi.nlm.nih.gov/genbank/) using the NCBI Nucleotide Basic Local Alignment Search Tool (BLASTN^**®**^) online algorithm (https://blast.ncbi.nlm.nih.gov/Blast.cgi), and ClustalW2 alignments with their closest matches were generated within Geneious R9 to determine nucleotide identities for the region of available sequence. PhyML 2.2.0 maximum likelihood phylogenetic trees for the herpesviruses were generated from ClustalW2 [59] nucleotide and amino acid sequence alignments using the general-time-reversible nucleotide substitution and Jones-Taylor-Thornton amino acid substitution models respectively, each with four substitution rate categories [60]. For the phylogenetic analysis of the family *Paramyxoviridae*, a ClustalW2 alignment [59] was used to draw a PhyML 2.2.0 maximum likelihood phylogenetic tree using the HKY85 nucleotide substitution model with four substitution rate categories [60]. For each of these trees the reliability of each branch was tested using 100 replicates in a bootstrap resampling analysis.

## Results

### PCR detection of viruses

Pooled choanal/tracheal and cloacal/intestinal swab samples from the study population detailed in S1 Table were screened for the presence of viruses from three families of RNA viruses, with the exception that samples from a wild powerful owl (*Ninox strenua*) swabbed at the very end of the sampling period were not included. No influenza A viruses or avian coronaviruses were detected from any of these 408 birds (0% prevalence; 95% C. I. 0–0.6%), while a single avian paramyxovirus was detected from a choanal swab from a wild musk lorikeet (*Glossopsitta concinna*) using the pan-paramyxovirus RT-PCR assay (0.25% prevalence; 95% C. I. 0–1.1%) (Table 1). While no avian paramyxoviruses were detected from the 110 captive birds tested in this study, there was not a statistically significant difference in the prevalence of avian paramyxovirus detection between wild and captive birds (p > 0.5; Fisher exact test).

**Table 1:** Detection of viruses by host order (number of birds from which virus detected/total number of birds tested).

Avian Order	Avian herpesviruses	Avian paramyxoviruses	Avian gamma-coronaviruses	Avian influenza viruses
Anseriformes	0/24	0/24	0/24	0/24
Columbiformes	0/35	0/35	0/35	0/35
Caprimulgiformes	1/35 –a novel avian herpesvirus detected from a tawny frogmouth (Podargidae; *Podargus strigoides*), tentatively designated *Podargid alphaherpesvirus 1*	0/35	0/35	0/35
Gruiformes	0/7	0/7	0/7	0/7
Charadriiformes	0/4	0/4	0/4	0/4
Procellariiformes	0/1	0/1	0/1	0/1
Pelecaniformes	0/13	0/13	0/13	0/13
Accipitriformes	0/6	0/6	0/6	0/6
Strigiformes	1/10 –*Columbid alphaherpesvirus 1* detected from a powerful owl (Strigidae; *Ninox strenua*)	0/9	0/9	0/9
Coraciiformes	0/27	0/27	0/27	0/27
Falconiformes	0/3	0/3	0/3	0/3
Psittaciformes	1/193 –a novel avian herpesvirus detected from a sulphur-crested cockatoo (Cacatuidae; *Cacatua galerita*), tentatively designated *Cacatuid alphaherpesvirus 1*	1/193 –a putative novel genotype of *Avian avulavirus 5* (APMV-5) detected from a musk lorikeet (Psittaculidae; *Glossopsitta concinna*), designated ‘unclassified avian avulavirus strain musk lorikeet/ Melbourne/ML22-141263/2014’	0/193	0/193
Passeriformes	0/51	0/51	0/51	0/51
**Total**	**3/409**	**1/408**	**0/408**	**0/408**

When DNA extracted from swab samples from the whole study population of 409 birds and separate liver swab samples from the 210 birds that had been sampled at necropsy (see S1 Table) was screened by the universal herpesvirus PCR assay [51], avian herpesvirus DNA was detected from three birds (0.7% prevalence; 95% C. I. 0.2–1.9%): a sulphur-crested cockatoo (*Cacatua galerita*), a tawny frogmouth (*Podargus strigoides*) and a powerful owl (Table 1). These three birds were all wildlife, with no avian herpesviruses being detected from the captive birds tested. However there was not a statistically significant difference in the prevalence of avian herpesvirus detection between wild and captive birds in this study (p > 0.5; Fisher exact test).

### Phylogenetic analysis of detected viruses

#### Detection of a known avian herpesvirus from a powerful owl

The 181 bp nucleotide sequence obtained from the powerful owl aligned with the DNA polymerase genes of strains of *Columbid alphaherpesvirus 1* (CoHV1) with 100% nucleotide identity [61–67]. Of the 210 liver swab samples screened by the universal herpesvirus PCR, only DNA extracted from the liver of the same powerful owl gave a strong positive band. A liver sample from this same individual owl also tested positive for CoHV1 in another study by Phalen, Alvarado, et al. [64]. The gross and histopathological lesions present in this owl were consistent with herpesviral inclusion body disease as described in Gailbreath and Lindsay Oaks [62]. Histopathology revealed extensive acute necrosis throughout the spleen, liver, pancreas and adrenal gland associated with eosinophilic to amphophilic intranuclear inclusion bodies in adjacent intact cells, with minimal associated inflammation. This female powerful owl had been found moribund on 1^st^ May 2015 in St Kilda, Victoria (37°87'17"S, 144°97'95"E), and died shortly after admission to the AWHC. This same powerful owl has been the subject of previously reported diagnostic investigations which revealed the presence of BFDV DNA in a sample of liver [21].

#### Detection of two novel avian herpesviruses from a tawny frogmouth and a sulphur-crested cockatoo

Two novel members of the family *Herpesviridae* were detected, one from DNA extracted from a choanal swab from a tawny frogmouth, and the other from DNA extracted from a tracheal swab collected from a sulphur-crested cockatoo. The closest matches to the 485 bp nucleotide sequence obtained from the tawny frogmouth swab were avian herpesviruses within the subfamily *Alphaherpesvirinae*, with highest (76%) nucleotide identity with the DNA polymerase genes of strains of CoHV1 [61, 63–65, 67]. It is unknown whether the tawny frogmouth is the natural host of this newly identified alphaherpesvirus, but lacking information to the contrary we have tentatively designated the virus *Podargid alphaherpesvirus 1* (PodHV1). The nucleotide sequence of the DNA polymerase gene fragment from this novel herpesvirus has been deposited in GenBank (accession number MF576272).

The tawny frogmouth had presented at the AWHC with a minor laceration and clinical signs suggestive of head trauma (weak, depressed and ataxic with blood in the oral cavity) on 27^th^ August 2015, but after a two week rehabilitation went on to fly off when released back where found in Badger Creek, Victoria (37°67'87"S, 145°53'99"E). The virus was not detected in DNA extracted from pooled choanal/tracheal and cloacal/intestinal swab samples from any of the other 34 tawny frogmouths tested (2.9% prevalence; 95% C. I. 0.3–12.6%), nor any of the other 82 avian species in this study. The clinical significance of this newly identified alphaherpesvirus is undetermined, but the tawny frogmouth showed no expression of clinical disease during its period of rehabilitation.

**Fig 1:**
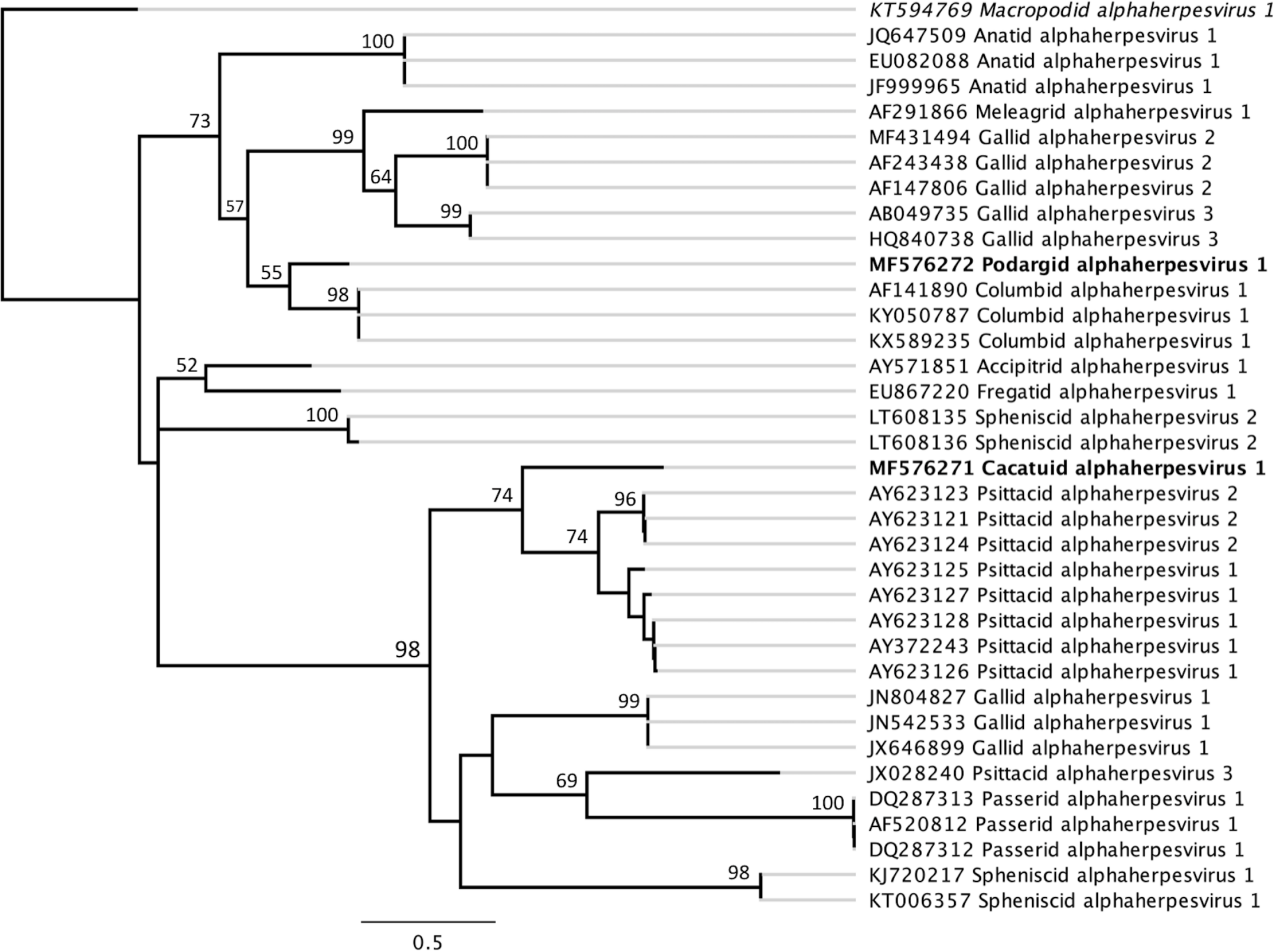
PhyML maximum likelihood phylogenetic tree of avian alphaherpesviruses. Generated from a ClustalW2 alignment [59] of the partial DNA polymerase gene sequences of ***Podargid alphaherpesvirus 1*** and ***Cacatuid alphaherpesvirus 1*** with published avian alphaherpesvirus nucleotide sequences available in GenBank, with the two novel alphaherpesviruses detected in this study highlighted in bold [60]. The GenBank accession numbers for sequences used are included in the tip labels, and *Macropodid alphaherpesvirus 1* (highlighted in italics) is included as an outgroup. Branching with greater than 50% support from 100 bootstrap replicates is indicated at major node points. The distances indicated by black horizontal lines correspond to genetic distances, with the scale bar representing nucleotide substitutions/site.

**Fig 2:**
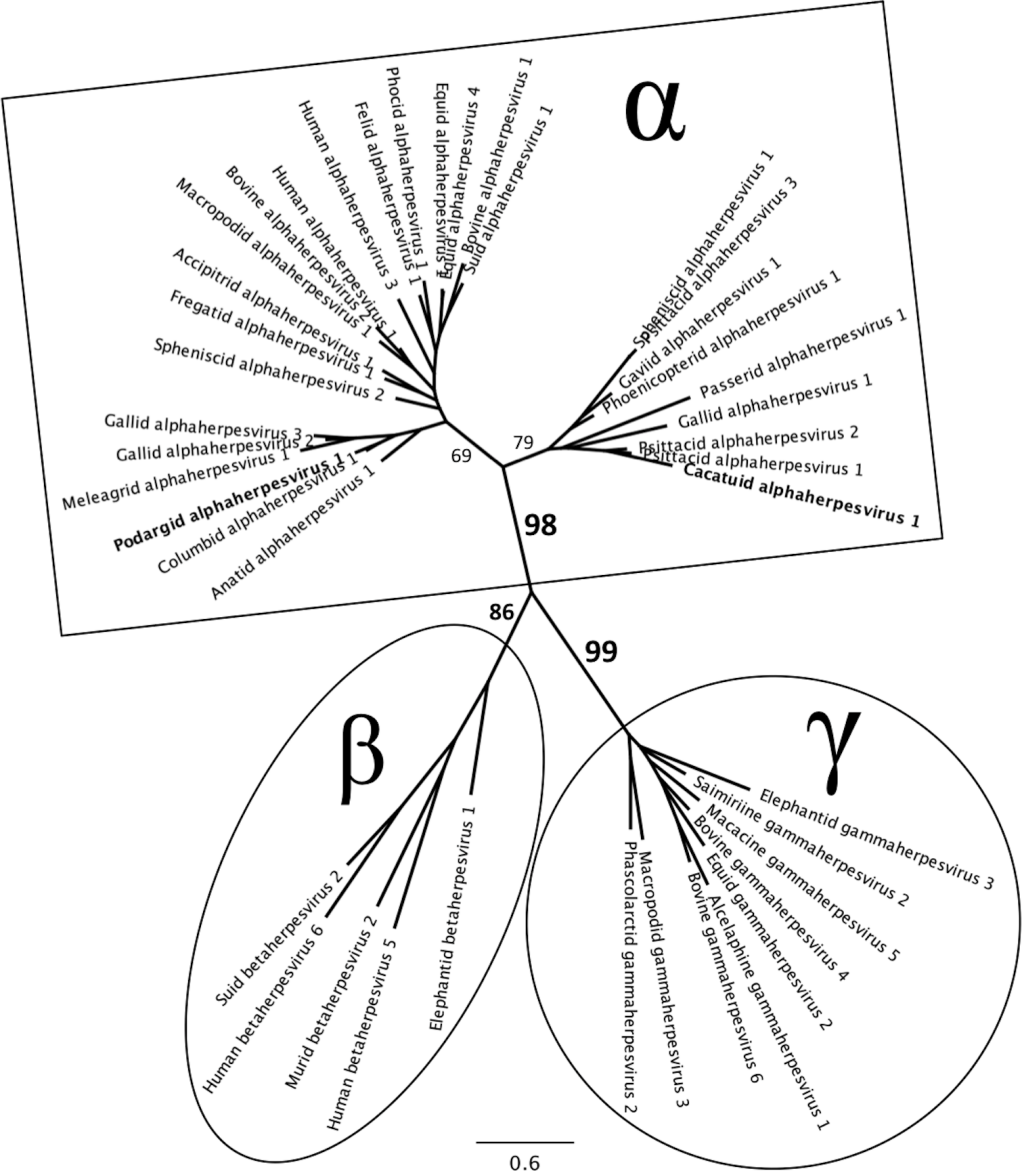
Unrooted maximum likelihood phylogenetic tree for the family *Herpesviridae*. Generated from a ClustalW2 alignment of amino acid translations of partial DNA polymerase gene sequences from 40 representative herpesviruses retrieved from GenBank from the three subfamilies: *Alphaherpesvirinae* (α), *Betaherpesvirinae* (β) and *Gammaherpesvirinae* (γ) from a range of host species, and including the two novel alphaherpesviruses detected in this study (highlighted in bold) [60]. The GenBank accession numbers for sequences used are as follows: *Accipitrid alphaherpesvirus 1* AY571851; *Alcelaphine gammaherpesvirus 1* AF005370; *Anatid alphaherpesvirus 1* EF643560; *Bovine alphaherpesvirus 1* X94677; *Bovine alphaherpesvirus 2* AF181249; *Bovine gammaherpesvirus 4* AF031811; *Bovine gammaherpesvirus 6* AF031808; ***Cacatuid alphaherpesvirus 1* MF576271**; *Columbid alphaherpesvirus 1* AF141890; *Elephantid betaherpesvirus 1* AF322977; *Elephantid gammaherpesvirus 3* DQ238845; *Equid alphaherpesvirus 1* KF434378; *Equid gammaherpesvirus 2* NC001650; *Equid alphaherpesvirus 4* KT324743; *Felid alphaherpesvirus 1* KR296657; *Fregatid alphaherpesvirus 1* EU867220; *Gallid alphaherpesvirus 1* NC006623; *Gallid alphaherpesvirus 2* AF147806; *Gallid alphaherpesvirus 3* HQ840738; *Gaviid alphaherpesvirus 1* GU130289; *Human alphaherpesvirus 1* HQ123098; *Human alphaherpesvirus 3* X04370; *Human betaherpesvirus 5* NC006273; *Human betaherpesvirus 6* X83413; *Macacine gammaherpesvirus 5* AF029302; *Macropodid alphaherpesvirus 1* NC029132; *Macropodid gammaherpesvirus 3* EF467663; *Meleagrid alphaherpesvirus 1* AF291866; *Murid betaherpesvirus 2* AY728086; *Passerid alphaherpesvirus 1* AF520812; *Phascolarctid gammaherpesvirus 2* JQ996387; *Phocid alphaherpesvirus 1* PHU92269; *Phoenicopterid alphaherpesvirus 1* KP244360; ***Podargid alphaherpesvirus 1* MF576272**; *Psittacid alphaherpesvirus 1* AY372243; *Psittacid alphaherpesvirus 2* AY623124; *Psittacid alphaherpesvirus 3* JX028240; *Saimiriine gammaherpesvirus 2* AJ410493; *Spheniscid alphaherpesvirus 1* KJ720217; *Spheniscid alphaherpesvirus 2* LT608135; *Suid alphaherpesvirus 1* BK001744; *Suid betaherpesvirus 2* AF268042. Percentage support from 100 bootstrap replicates is indicated at major branch points. The scale bar represents amino acid substitutions/site.

The closest match to the 489 bp nucleotide sequence from the sulphur-crested cockatoo swab was the DNA polymerase gene of PsHV1 (GenBank accession number AY372243), with 66.5% nucleotide identity [68]. This sequence also had 65% nucleotide identity with the limited DNA polymerase gene sequence available (266 bp) for *Psittacid alphaherpesvirus 2* (PsHV2), which has been detected in African grey parrots (*Psittacus erithacus*) and a blue and gold macaw (*Ara ararauna*) in the USA [22, 69]. The phylogenetic trees (Figs 1 and 2) suggest that this novel herpesvirus belongs to the subfamily *Alphaherpesvirinae*, and we tentatively propose the name *Cacatuid alphaherpesvirus 1* (CacHV1) after the host family in which it was found. The nucleotide sequence of the DNA polymerase gene fragment from this novel herpesvirus has been deposited in GenBank (accession number MF576271).

The female sulphur-crested cockatoo had been found unable to fly in Lilydale, Victoria (37°75'73"S, 145°37'20"E) on 17^th^ September 2015, presenting emaciated and weak with haemorrhagic enteritis and clinical signs suggestive of beak and feather disease virus (BFDV) infection. This cockatoo tested positive for BFDV when DNA extracted from a liver sample collected at post-mortem was used as template in a BFDV PCR assay [21, 70]. Histopathological examination of samples of liver, spleen, kidney, lung, heart, gastrointestinal tract, pancreas and ovarian tissue collected at necropsy revealed chronic enteritis with unidentified piriform protozoa in intestinal crypts and foci of mucosal and crypt necrosis with interstitial fibrosis in the submucosa of the proximal small intestine, along with a significant overgrowth of *Macrorhabdus ornithogaster* in the ventriculus. It is presumed that the protozoan and *Macrorhabdus* overgrowth were secondary to immunosuppression caused by BFDV infection, but it is unknown what role the newly identified alphaherpesvirus may have been playing in this emaciated and very unwell bird. No inclusion bodies, inflammation in the lungs nor any other sign of herpesvirus infection were seen in any of the tissues examined histologically. CacHV1 was only detected from one of the 27 sulphur-crested cockatoos tested in this study (3.7% prevalence; 95% C. I. 0.4–16.0%), and was not detected from any of the other 54 Cacatuids tested (1.8% prevalence; 95% C. I. 0.2–8.2%).

#### Detection of an avian paramyxovirus from a wild musk lorikeet

**Fig 3:**
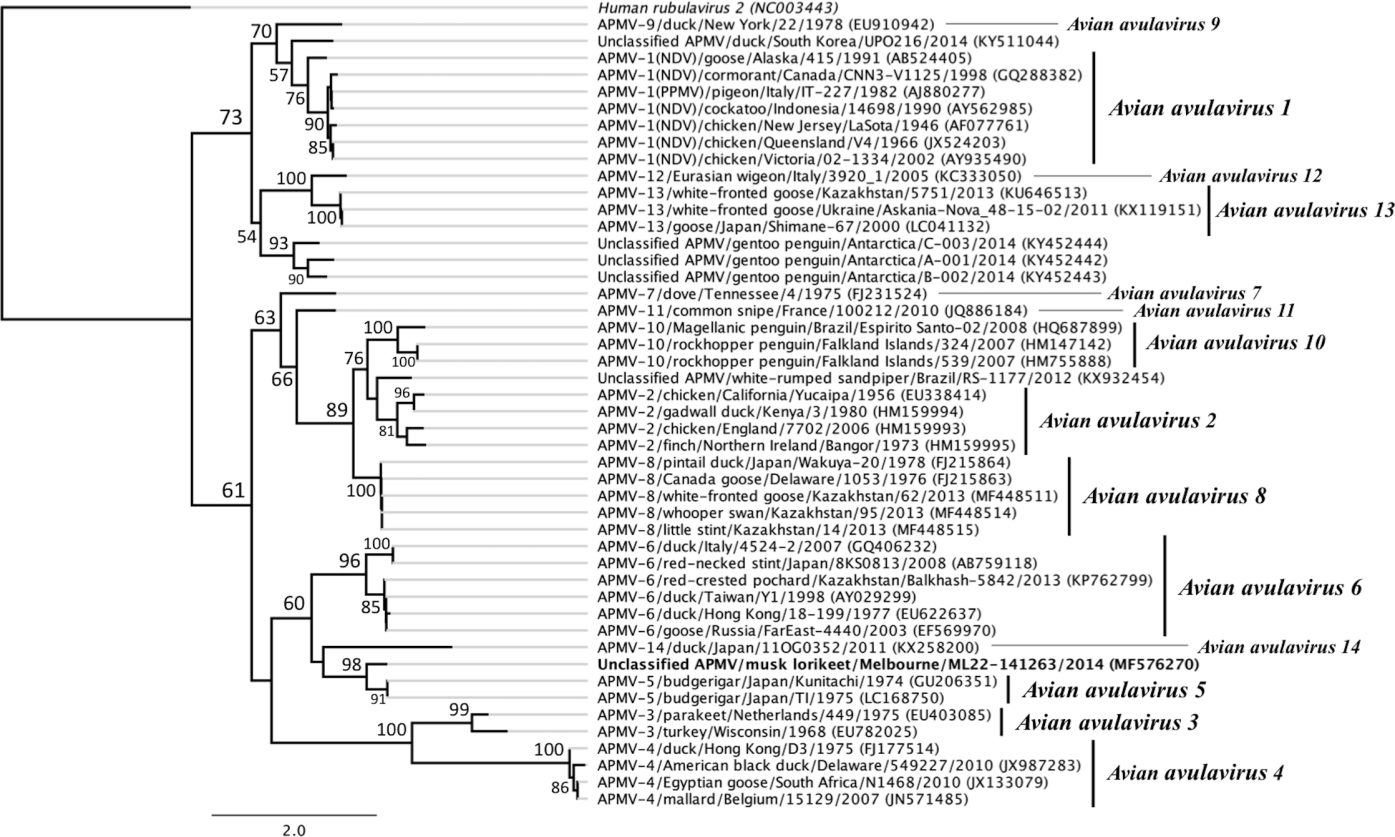
Phylogenetic tree for the genus *avulvirus* of the family *Paramyxoviridae* based on partial RNA-dependent RNA polymerase gene sequences. Maximum likelihood phylogenetic tree constructed using PhyML from a ClustalW2 alignment of the partial RNA-dependent RNA polymerase (large polymerase or L protein) gene sequences of the avian avulavirus detected from a musk lorikeet (*Glossopsitta concinna*) in this study (unclassified avian avulavirus strain musk lorikeet/Melbourne/ML22-141263/2014; highlighted in bold) and published paramyxovirus sequences retrieved from GenBank [60]. The GenBank accession numbers for sequences used are indicated in brackets in the tip labels. *Human rubulavirus 2* (highlighted in italics) is included as an outgroup. Branching with greater than 50% support from 100 bootstrap replicates is indicated at major node points. The distances indicated by black horizontal lines correspond to genetic distances, with the scale bar representing nucleotide substitutions/site.

The musk lorikeet had been found unable to fly in Bundoora Park (37°42'28"S, 145°02'42"E) in December 2014, and presented to the AWHC with an old fracture of the right coracoid, on the day of sample collection. After a successful four-week rehabilitation, during which the lorikeet ate well, gained bodyweight and showed no signs of ill health, the bird was released back where it was found. On BLAST analysis, the 603 bp sequence fragment of the paramyxovirus RNA-dependent RNA polymerase (L protein) gene obtained from this musk lorikeet did not match any currently known paramyxovirus, but had highest (77.4–77.6%) nucleotide sequence identity with strains of *Avian avulavirus 5* (GenBank accession numbers GU206351 and LC168750) [34, 71]. The partial RNA-dependent RNA polymerase gene sequence of the avian avulavirus detected in this study (strain musk lorikeet/Melbourne/ML22-141263/2014) has been deposited in GenBank (accession number MF576270). Phylogenetic analysis suggests that this avulavirus strain belongs to the serotype *Avian avulavirus 5*, and perhaps represents a novel genotype or genetic subgroup within this serotype (Fig 3). Paramyxovirus RNA was not detected from any of the other three wild musk lorikeets tested in this study, giving a prevalence of 25% (95% C. I. 2.9–71.6%) for the species.

## Discussion

A very low prevalence of viruses was detected in the birds examined in this study. Sampling birds presented to veterinary hospitals, such as the AWHC, is a useful approach to studying viruses in wild birds. However, this approach does involve an inherently biased population sample (for example a bias towards sick or injured birds, particular locations and common urban species) which should be considered when these results are interpreted, or compared to other studies. It is likely that the sample population in this present study, particularly the low numbers of Anseriformes and Charadriiformes (28 of the 408 birds tested) contributed to the absence or low prevalence of detection of AIVs, APMVs and AvCoVs, as previous studies have shown that these viruses are present mainly in the orders Anseriformes and Charadriiformes [3, 15, 36, 38, 46, 72–74]. The risk-based Australian National Avian Influenza Wild Bird Surveillance Program detected a low prevalence (1.7%) of AIVs in 66,987 Australian wild birds between 2005 and 2012 using a study population containing over 90% Anseriformes or Charadriiformes [46, 47]. This contrasts with the results from this study where AIVs were not detected in any of the birds sampled. Together these results suggest that, although low pathogenic AIVs are circulating at low prevalence in wild birds (particularly those belonging to the orders Anseriformes and Charadriiformes) in Australia [46, 47], there is a low risk of AIV infection in birds presenting for veterinary care at the AWHC.

Studies of avian coronaviruses in wild birds in other countries, using the same primers [54] as the present study, have detected AvCoVs at a higher prevalence than was determined in this present study, including 13% and 3.5% of wild bird samples tested in studies in Brazil and Poland, respectively [75, 76]. These primers detect gammacoronaviruses of the IBV lineage, but do not appear to detect deltacoronaviruses. Whilst the present study was interested in investigating an hypothesised wild bird AvCoV origin of genetic components of variant IBV strains infecting commercial poultry in Australia [48, 50], future use of a pan-*Coronaviridae* PCR assay to test Australian wild bird samples for AvCoVs would be useful for gaining a more general understanding of AvCoVs in Australia. Future studies focused on elucidating the origin of variant IBV in Australian poultry may be best focused on species of wild birds known to be in closest contact with commercial poultry, such as introduced (feral) passerine birds that commonly live in close proximity to humans and domestic animal production facilities, like European starlings (*Sturnus vulgaris*) and house sparrows (*Passer domesticus*) [77, 78].

Our results showing a very low prevalence (0.25%) of APMVs in wild birds in Victoria are consistent with the results of other similar Australian studies [15, 79, 80]. The paramyxovirus detected in a musk lorikeet had highest nucleotide sequence identity to *Avian avulavirus 5*, which had previously only been isolated from budgerigars [25, 32–34, 71], and for which no sequence information from Australia is available. Budgerigars and musk lorikeets both belong to the family Psittaculidae within the avian order Psittaciformes. APMV-5 has been detected in epizootic outbreaks among captive budgerigar flocks in Queensland, Australia and Japan in the 1970s and in the UK in 1993, with high mortality and clinical signs including depression, dyspnoea, diarrhoea, vomiting and torticollis [32–34]. Of the currently recognized avian avulavirus serotypes, only Newcastle disease virus and APMV-5 have been associated with 100% mortality [34]. The clinical significance of the avulavirus strain detected from the musk lorikeet in the present study is undetermined, but the bird showed no clinical disease during rehabilitation.

It is possible that the APMV strain detected in this study represents an avirulent genotype of APMV-5 that may be circulating amongst wild psittacines in Australia. The complete genome sequences of strains of other avian paramyxoviruses have provided evidence for the existence of genetic subgroups within their respective serotypes [30, 81, 82]. Analysis of the same region of the RNA polymerase gene for which we have sequence information in the present study shows the different subgroups of APMV-2, APMV-3 and APMV-6 have 72.7–78.2%, 73.1% and 75.5–76.2% nucleotide sequence identity respectively within their serotypes [30, 81, 82]. By comparison, the APMV strain detected from the musk lorikeet in this study has 77.4–77.6% nucleotide identity with the APMV-5 sequences obtained from budgerigars in Japan in the 1970s [34, 71]. This suggests that the avian avulavirus detected in this study represents a novel genetic subgroup or genotype of the serotype *Avian avulavirus 5*, but this putative classification can only be confirmed if the virus can be isolated in the future for antigen-based testing and more extensive genome sequencing. Formal classification of APMV strain musk lorikeet/Melbourne/ML22-141263/2014 will require more genomic sequence information than the 603 bp of the RNA polymerase gene that was obtained in the current study. The clinical significance of this possible novel APMV-5 genotype in other Psittaciformes, such as those in involved in threatened species recovery programs, is also undetermined.

Despite 193 birds belonging to the order Psittaciformes being tested in this study, which included seven orange-bellied parrots from Healesville Sanctuary’s collection, neither *Avian avulavirus 3* nor *Psittacid alphaherpesvirus 1* were detected. Many species of Australian parrots are susceptible to APMV-3 and/or PsHV1 infection and disease [26–28, 83], so the potential establishment of both viruses in Australia poses significant risks to Australian Psittaciformes and their conservation [23], particularly to breeding programs for threatened species, such as the orange-bellied parrot captive breeding program at Healesville Sanctuary. Both APMV-3 and PsHV1 have been reported in numerous Australian psittacine species held in captivity overseas, and have caused significant mortality events in avicultural collections in other countries [23, 26–28, 83]. APMV-3 has not yet been reported in Australia [25–31], while PsHV1 has not been reported in wild Psittaciformes in Australia [23]. However it is known that PsHV1 entered Australia in captive green-winged macaws and is likely to present at a low prevalence in some avian collections, where latently infected parrots may be potential sources of virus dissemination [22]. Ongoing surveillance of both wild and captive Psittaciformes for these viruses is vital for detecting and managing potential outbreaks of these pathogens should they be introduced into Australia’s avifauna. Our results showing an absence of PsHV1 in the sampled birds are consistent with results from a serologic survey for PsHV1 in Australian psittacines from New South Wales, Tasmania, Victoria and Western Australia, where all 411 wild and captive birds tested were negative for the presence of neutralizing antibody to PsHV1 [84].

Three avian herpesviruses were detected in the present study: *Columbid alphaherpesvirus 1* in a powerful owl and two novel viruses in a sulphur-crested cockatoo and a tawny frogmouth, respectively. CoHV1 infection had previously been documented in three species of native birds of prey in Australia: powerful owls, barking owls (*Ninox connivens*) and an Australian hobby (*Falco longipennis*) [65]. The virus causes herpesviral inclusion body disease or inclusion body hepatitis in birds of prey (considered aberrant hosts of this virus) when they ingest infected natural hosts of CoHV1 (rock doves or rock pigeons (*Columba livia*)) [62, 64–66, 85, 86]. A recent study has suggested that CoHV1 is enzootic in feral pigeon flocks in Australia, with CoHV1 DNA detected in samples from 81% of 53 feral pigeons from five flocks in Victoria and New South Wales [64]. This recent study also tested oral swabs from 18 Australian native columbids by PCR and all were found to be negative for CoHV1 DNA [64]. The present study tested six wild native Australian pigeons and 28 captive native Australian pigeons and doves. CoHV1 DNA was not detected from any of these birds, suggesting that native columbids are not likely to be a reservoir of CoHV1 infection in Australia.

The two novel herpesviruses detected in this study, tentatively designated CacHV1 and PodHV1, are interesting additions to our knowledge of herpesviruses in avian species. The genetic identification of previously undiscovered avian viruses is essential for mapping disease spread by the international trade in birds, managing biosecurity and disease outbreaks in aviaries, and managing threatened birds being bred for release for conservation of their species [87]. The 485 bp sequence obtained from the tawny frogmouth and the 489 bp sequence obtained from the sulphur-crested cockatoo showed 76% and 66.5% nucleotide identities to the most genetically similar known herpesviruses (CoHV1 and PsHV1, respectively). This level of identity in this highly conserved region of the herpesvirus DNA polymerase gene is sufficient to indicate that the viruses are new species. As a comparison, there is 82% nucleotide identity at this same region between the distinct but closely related species *Equid alphaherpesvirus 1* and *4* (EHV1 and EHV4, respectively), but there is 100% nucleotide identity at this same region between different isolates of EHV1 and 100% identity between different isolates of EHV4 [88]. Serological cross neutralization has been reported between closely related alphaherpesviruses [89], thus the presence of the newly detected CacHV1 should be considered if any future serological surveys for PsHV1 return positive results. The phylogenetic analyses of sequence data in the present study is sufficient to place the newly detected viruses in the subfamily *Alphaherpesvirinae* (Figs 1 and 2), but insufficient to classify PodHV1 and CacHV1 beyond subfamily level. The clinical significance of these viruses remains undetermined, and more extensive genome sequencing will be required for the formal naming, description and classification of these previously unknown avian herpesviruses through the International Committee on Taxonomy of Viruses.

This study has contributed valuable information regarding viruses present (and absent) in Australian wild birds and the level of potential risk to animal and human health, and to threatened avian species in Australia. Whilst it is interesting that no avian herpesviruses nor paramyxoviruses were detected from the captive birds tested in this study, there was no significant difference in the prevalence of detection of avian viruses between the wild and captive bird study populations. The detection of two previously unknown viruses has contributed to our knowledge of avian viral communities and their diversity in Australia. Future studies incorporating deep sequencing methods could be expected to possibly uncover more avian viruses and make further contributions to our understanding of avian virodiversity.

## Supporting information

S1 Table Species composition of study population by taxonomic group. Wild birds were presented to the AWHC at Healesville Sanctuary for veterinary care. Captive birds were part of the Healesville Sanctuary collection.
(DOCX)
