# Supplementary material for: Avian viral surveillance in Victoria, Australia, and detection of two novel avian herpesviruses
Source: PLoS One. 2018 Mar 23;13(3):e0194457. doi: 10.1371/journal.pone.0194457 (PMC5865735; doi:10.1371/journal.pone.0194457)
Supplement: S1 Table — Wild birds were presented to the AWHC at Healesville Sanctuary for veterinary care. Captive birds were part of the Healesville Sanctuary collection. (DOCX) [file pone.0194457.s001.docx]

**S1 Table. Species Composition of Study Population by Taxonomic Group.**

| **Order** | **Family** | **Bird species**  **(common name)** | **Scientific name** | **Wild** ^a^ **birds swabbed live** | **Captive** ^b^ **birds swabbed live** | **Wild** ^a^ **birds sampled at necropsy** | **Captive** ^b^ **birds sampled at necropsy** | **Total wild** ^a^ **birds** | **Total captive** ^b^ **birds** | **TOTAL** |
| --- | --- | --- | --- | --- | --- | --- | --- | --- | --- | --- |
| Anseriformes  n = 24 | Anatidae  n = 24 | Black swan | *Cygnus atratus* | 1 |  |  |  | **1** |  | **1** |
|  |  | Australian shelduck | *Tadorna tadornoides* |  |  | 1 |  | **1** |  | **1** |
|  |  | Hardhead | *Aythya australis* |  |  |  | 1 |  | **1** | **1** |
|  |  | Pacific black duck | *Anas superciliosa* | 1 |  | 4 |  | **5** |  | **5** |
|  |  | Chestnut teal | *Anas castanea* | 1 |  | 1 | 3 | **2** | **3** | **5** |
|  |  | Australian wood duck | *Chenonetta jubata* | 6 | 1 | 4 |  | **10** | **1** | **11** |
| Columbiformes  n = 35 | Columbidae  n = 35 | White-headed pigeon | *Columba leucomela* |  | 12 |  |  |  | **12** | **12** |
|  |  | Brown cuckoo-dove | *Macropygia phasianella* |  | 1 |  |  |  | **1** | **1** |
|  |  | Wonga pigeon | *Leucosarcia melanoleuca* |  | 3 |  |  |  | **3** | **3** |
|  |  | Common bronzewing | *Phaps chalcoptera* | 2 |  | 2 |  | **4** |  | **4** |
|  |  | Brush bronzewing | *Phaps elegans* |  | 5 |  |  |  | **5** | **5** |
|  |  | Crested pigeon | *Ocyphaps lophotes* |  |  | 2 |  | **2** |  | **2** |
|  |  | Peaceful dove | *Geopelia placida* |  |  |  | 1 |  | **1** | **1** |
|  |  | Brown-capped emerald dove | *Chalcophaps longirostris* |  | 3 |  |  |  | **3** | **3** |
|  |  | Rose-crowned fruit dove | *Ptilinopus regina* |  | 3 |  |  |  | **3** | **3** |
|  |  | Spotted dove (introduced) | *Spilopelia chinensis* |  |  | 1 |  | **1** |  | **1** |
| Caprimulgiformes  n = 35 | Podargidae  n = 35 | Tawny frogmouth | *Podargus strigoides* | 12 |  | 23 |  | **35** |  | **35** |
| Gruiformes  n = 7 | Rallidae  n = 7 | Buff-banded rail | *Hypotaenidia philippensis* |  | 1 |  |  |  | **1** | **1** |
|  |  | Purple swamphen | *Porphyrio porphyrio* |  |  | 5 |  | **5** |  | **5** |
|  |  | Eurasian coot | *Fulica atra* | 1 |  |  |  | **1** |  | **1** |
| Charadriiformes  n = 4 | Burhinidae  n = 1 | Bush stone-curlew | *Burhinus grallarius* |  | 1 |  |  |  | **1** | **1** |
|  | Recurvirostridae  n = 1 | Pied stilt | *Himantopus himantopus* |  | 1 |  |  |  | **1** | **1** |
|  | Charadriidae  n = 2 | Hooded plover | *Thinornis cucullatus* |  |  | 1 |  | **1** |  | **1** |
|  |  | Inland dotterel | *Peltohyas australis* |  | 1 |  |  |  | **1** | **1** |
| Procellariiformes  n = 1 | Procellariidae  n = 1 | Short-tailed shearwater | *Ardenna tenuirostris* |  |  | 1 |  | **1** |  | **1** |
| Pelecaniformes  n = 13 | Ardeidae  n = 6 | Pied heron | *Egretta picata* |  | 1 |  |  |  | **1** | **1** |
|  |  | White-faced heron | *Egretta novaehollandiae* |  | 4 |  | 1 |  | **5** | **5** |
|  | Threskiornithidae  n = 7 | Australian white ibis | *Threskiornis moluccus* |  |  | 5 |  | **5** |  | **5** |
|  |  | Royal spoonbill | *Platalea regia* |  | 1 |  |  |  | **1** | **1** |
|  |  | Glossy ibis | *Plegadis falcinellus* |  | 1 |  |  |  | **1** | **1** |
| Accipitriformes  n = 6 | Accipitridae  n = 6 | Black-shouldered kite | *Elanus axillaris* | 1 |  |  |  | **1** |  | **1** |
|  |  | Wedge-tailed eagle | *Aquila audax* | 2 |  | 1 |  | **3** |  | **3** |
|  |  | Brown goshawk | *Accipiter fasciatus* |  |  | 1 |  | **1** |  | **1** |
|  |  | Black kite | *Milvus migrans* | 1 |  |  |  | **1** |  | **1** |
| Strigiformes  n = 10 | Tytonidae  n = 1 | Barn owl | *Tyto alba* |  |  | 1 |  | **1** |  | **1** |
|  | Strigidae  n = 9 | Powerful owl | *Ninox strenua* | 2 |  | 1 |  | **3** |  | **3** |
|  |  | Southern boobook | *Ninox boobook* |  |  | 6 |  | **6** |  | **6** |
| Coraciiformes  n = 27 | Alcedinidae  n = 27 | Sacred kingfisher | *Todiramphus sanctus* |  | 1 | 1 |  | **1** | **1** | **2** |
|  |  | Laughing kookaburra | *Dacelo novaeguineae* | 11 | 1 | 13 |  | **24** | **1** | **25** |
| Falconiformes  n = 3 | Falconidae  n = 3 | Australian hobby | *Falco longipennis* | 1 |  |  |  | **1** |  | **1** |
|  |  | Peregrine falcon | *Falco peregrinus* | 1 |  | 1 |  | **2** |  | **2** |
| Psittaciformes  n = 193 | Cacatuidae  n = 55 | Cockatiel | *Nymphicus hollandicus* |  | 1 |  | 1 |  | **2** | **2** |
|  |  | Red-tailed black cockatoo | *Calyptorhynchus banksii* |  | 1 |  |  |  | **1** | **1** |
|  |  | Yellow-tailed black cockatoo | *Zanda funerea* |  |  | 1 |  | **1** |  | **1** |
|  |  | Gang-gang cockatoo | *Callocephalon fimbriatum* | 2 | 1 | 3 |  | **5** | **1** | **6** |
|  |  | Galah | *Eolophus roseicapilla* | 4 |  | 7 |  | **11** |  | **11** |
|  |  | Long-billed corella | *Cacatua tenuirostris* | 1 | 1 | 1 |  | **2** | **1** | **3** |
|  |  | Little corella | *Cacatua sanguinea* | 3 |  | 1 |  | **4** |  | **4** |
|  |  | Sulphur-crested cockatoo | *Cacatua galerita* | 10 |  | 17 |  | **27** |  | **27** |
|  | Psittaculidae  n = 138 | Superb parrot | *Polytelis swainsonii* |  | 4 |  |  |  | **4** | **4** |
|  |  | Princess parrot | *Polytelis alexandrae* |  | 1 |  |  |  | **1** | **1** |
|  |  | Australian king parrot | *Alisterus scapularis* | 8 | 2 | 28 | 2 | **36** | **4** | **40** |
|  |  | Golden-shouldered parrot | *Psephotellus chrysopterygius* |  | 2 |  |  |  | **2** | **2** |
|  |  | Crimson rosella | *Platycercus elegans* | 12 | 6 | 18 | 2 | **30** | **8** | **38** |
|  |  | Eastern rosella | *Platycercus eximius* | 1 |  | 11 |  | **12** |  | **12** |
|  |  | Swift parrot | *Lathamus discolor* |  | 1 |  |  |  | **1** | **1** |
|  |  | Orange-bellied parrot | *Neophema chrysogaster* |  | 6 |  | 1 |  | **7** | **7** |
|  |  | Scarlet-chested parrot | *Neophema splendida* |  |  |  | 1 |  | **1** | **1** |
|  |  | Musk lorikeet | *Glossopsitta concinna* | 2 |  | 2 |  | **4** |  | **4** |
|  |  | Rainbow lorikeet | *Trichoglossus moluccanus* | 8 | 4 | 5 |  | **13** | **4** | **17** |
|  |  | Scaly-breasted lorikeet | *Trichoglossus chlorolepidotus* |  |  |  | 4 |  | **4** | **4** |
|  |  | Budgerigar | *Melopsittacus undulates* |  | 7 |  |  |  | **7** | **7** |
| Passeriformes  n = 51 | Menuridae n = 3 | Superb lyrebird | *Menura novaehollandiae* | 2 |  | 1 |  | **3** |  | **3** |
|  | Ptilonorhynchidae  n = 2 | Satin bowerbird | *Ptilonorhynchus violaceus* |  | 1 | 1 |  | **1** | **1** | **2** |
|  | Meliphagidae  n = 7 | White-eared honeyeater | *Nesoptilotis leucotis* |  | 1 |  |  |  | **1** | **1** |
|  |  | Red wattlebird | *Anthochaera carunculata* | 3 |  |  |  | **3** |  | **3** |
|  |  | Yellow-tufted honeyeater | *Lichenostomus melanops* |  | 2 |  | 1 |  | **3** | **3** |
|  | Pardalotidae n = 1 | Striated pardalote | *Pardalotus striatus* |  |  | 1 |  | **1** |  | **1** |
|  | Acanthizidae n = 1 | Brown thornbill | *Acanthiza pusilla* |  |  | 1 |  | **1** |  | **1** |
|  | Campephagidae n = 1 | Black-faced cuckoo-shrike | *Coracina novaehollandiae* |  | 1 |  |  |  | **1** | **1** |
|  | Pachycephalidae n = 1 | Grey shrike-thrush | *Colluricincla harmonica* |  |  | 1 |  | **1** |  | **1** |
|  | Psophodidae n = 1 | Eastern whipbird | *Psophodes olivaceus* |  | 1 |  |  |  | **1** | **1** |
|  | Artamidae  n = 21 | Pied currawong | *Strepera graculina* |  |  | 1 |  | **1** |  | **1** |
|  |  | Australian magpie | *Gymnorhina tibicen* | 6 |  | 11 |  | **17** |  | **17** |
|  |  | White-browed woodswallow | *Artamus superciliosus* |  | 3 |  |  |  | **3** | **3** |
|  | Rhipiduridae n = 1 | Willie wagtail | *Rhipidura leucophrys* |  | 1 |  |  |  | **1** | **1** |
|  | Corvidae n = 4 | Australian raven | *Corvus coronoides* | 1 |  | 3 |  | **4** |  | **4** |
|  | Monarchidae n = 1 | Magpie lark | *Grallina cyanoleuca* |  | 1 |  |  |  | **1** | **1** |
|  | Estrildidae  n = 4 | Chestnut-breasted mannikin | *Lonchura castaneothorax* |  | 1 |  |  |  | **1** | **1** |
|  |  | Diamond firetail | *Stagonopleura guttata* |  | 2 |  |  |  | **2** | **2** |
|  |  | Red-browed finch | *Neochmia temporalis* |  |  | 1 |  | **1** |  | **1** |
|  | Turdidae  n = 3 | Bassian thrush | *Zoothera lunulata* | 1 |  | 1 |  | **2** |  | **2** |
|  |  | Eurasian blackbird (introduced) | *Turdus merula* |  |  | 1 |  | **1** |  | **1** |
| **TOTALS** | | **83 bird species** | | **107** | **92** | **192** | **18** | **299 wild** ^a^ **birds** | **110 captive** ^b^ **birds** | **409 BIRDS** |
| ^a^ Wild birds were presented to the AWHC at Healesville Sanctuary for veterinary care  ^b^ Captive birds were part of the Healesville Sanctuary collection | | | | **199 birds swabbed live** | | **210 birds sampled at necropsy**  **(swabs + liver sample collected)** | |  |  |  |
